# Weight and Height Percentiles For 0-84-Month-Old Children in Kayseri - A Central Anatolian City in Turkey

**DOI:** 10.4274/jcrpe.452

**Published:** 2011-12-06

**Authors:** Canan Altunay, Meda Kondolot, Serpil Poyrazoğlu, Ahmet Öztürk, Mümtaz M. Mazıcıoğlu, Selim Kurtoğlu

**Affiliations:** 1 Erciyes University Faculty of Medicine, Department of Pediatrics, Kayseri, Turkey; 2 Erciyes University Faculty of Medicine, Department of Pediatrics, Unit of Social Pediatrics, Kayseri, Turkey; 3 Erciyes University Faculty of Medicine, Department of Public Health, Kayseri, Turkey; 4 Erciyes University Faculty of Medicine, Department of Biostatistics, Kayseri, Turkey; 5 Erciyes University Faculty of Medicine, Department of Family Medicine, Kayseri, Turkey; 6 Erciyes University Faculty of Medicine, Department of Pediatric Endocrinology, Kayseri, Turkey; +90 352 438 00 76+90 352 437 58 25selimk@erciyes.edu.tr Erciyes University Faculty of Medicine, Department of Pediatric Endocrinology, Kayseri, Turkey

**Keywords:** weight, height, infant, toddler, percentiles, Turkey

## Abstract

**Objective:** The aim of this study was to present weight and height percentiles for Turkish children aged 0-84 months residing in Kayseri, Turkey and to compare these findings with national references and international standards.

**Methods:** We used the data from the Anthropometry of Turkish Children aged 0-6 years (ATCA-06) study. This cross-sectional study conducted in Kayseri/Turkey between September 2009 and May 2010 included 2963 children (1491 girls, 1472 boys) aged 0-84 months. The centile curves were constructed using the LMS method.

**Results:** The 3rd, 5th, 10th, 15th, 25th, 50th, 75th, 85th, 90th, 95th, and 97th percentiles and the LMS values for boys and girls were constructed. The 50th percentiles for weight and height of the children were compared with world health organization (WHO) standards and national data. Height and weight values in Kayseri children were lower than WHO standards and Istanbul references in the first year of life. At ages 1 to 4, weight values in both genders and height in boys were slightly higher than the national and international standards. Starting at age 4 years, the weight percentiles of Kayseri children were strikingly higher compared to the national and international standards and the boys were also taller.

**Conclusions:** This study provides cross-sectional data for weight and height percentiles of Turkish children aged 0-84 months residing in Kayseri. These data reflect the growth status of healthy Kayseri children and also indicate that these children may be more prone to obesity than the Istanbul children. Since the above-mentioned data illustrate the current growth status of this population, we believe that they will serve as a basis for monitoring future trends.

**Conflict of interest:**None declared.

## INTRODUCTION

The physical growth of infants and children is an important indicator of health and well-being. Growth charts have been used to assess whether a child is receiving adequate nutrition and to screen for potentially inadequate growth that might be a sign of adverse health conditions (1,2,3,4,5,6). The world health organization (WHO) Multicentre Growth Reference Study (MGRS) data demonstrate that healthy children from around the world, who are raised in healthy environments and follow recommended feeding practices, have very similar patterns of growth in the first 5 years of life (5). However, there is still a considerable variation across populations in height and weight, which is the result of genetic diversity, exposition to vastly different environmental factors, and differences in socioeconomic status (7,8). Because of these differences, it may be better that each country has its own growth references (9,10,11). There may be some differences even within the same population or within similar geographic locations (12,13,14). While longitudinal studies are recommended to monitor child growth, cross-sectional studies have the advantage of reflecting the current status of the population. They also serve to demonstrate changes in growth references over time (secular changes). 

In this cross-sectional study, we constructed weight and height percentiles for Turkish children aged 0-84 months living in a large city of Central Anatolia in Turkey. We also compared these percentiles with previously reported national references and with international standards. 

## MATERIALS AND METHODS

We used data from the Anthropometry of Turkish Children aged 0-6 years (ATCA-06) study that was conducted between September 2009 and May 2010. This cross-sectional study was conducted in Kayseri province which has more than 1 200 000 inhabitants and is a leading industrial trade center in Turkey. 

**Subjects and Sampling**

The primary sampling units were Family Health Centers (FHC) located in the city center and suburbs of Kayseri. Firstly, children aged 0-84 months were selected from 21 FHC and stratified according to the socioeconomic status of their parents. The data provided by the local health authority were used to group families into low, medium, and high socioeconomic levels, based on their income. Accordingly, 17.5% of the families were in the low, 58.4% were in the medium and 24.1% were in the high socioeconomic level brackets. The sample size was calculated as 3200. The families were invited to ASM by district midwives and we were able to reach 3094 children to participate in the study. Premature and low birth weight infants, multiple births, and children who had any known chronic or serious illness or malnutrition were excluded (n=131). A total of 2963 children (1491 girls, 1472 boys) aged 0-84 months were included in the study, with 1468 measurements in boys and 1491 measurements in girls for weight as well as 1472 measurements in boys and 1491 measurements in girls for height. The study protocol was approved by the Ethics Committee at Erciyes University School of Medicine and individual consent was taken from parents. 

**Anthropometric Techniques**

All measurements were performed by two trained health technicians. Weight and height were measured twice and the average for each was recorded. Weight was measured with an electronic digital scale (Seca 354; accurate to 10 g) for children aged 0-24 months in a dry diaper without clothing. In children older than 2 years, weight was measured with a standard beam balance scale (Tefal Ultraslim, France; accurate to 100 g) with children wearing only underwear. Length for children younger than 2 years was measured by two examiners (one to position the child) with the child supine on a measuring board. Height for older children was determined to the nearest 1 mm with a portable stadiometer in the standing upright position without shoes, with hips and shoulders perpendicular to the central axis, heels against the footboard, knees together, arms hanging loosely at the sides and the horizontal board of the stadiometer touching the head. The portable scales and stadiometers were calibrated daily. 

**Statistical Analysis**

Construction of the centiles for 0-84 months was performed with the LMS Chart Maker Pro version 2.3 software program (The Institute of Child Health, London), which fits smooth centiles to reference data using the LMS method (Cole and Green 1992). The percentile curves were constructed by Microsoft Office Excel version 2003. Inter-observer correlation coefficients were ≥0.98. 

## RESULTS

A total of 131 (4%) children were excluded because of disorders that may interfere with growth and development, thus, the final number of subjects included in the study was 2963. All infants and children included in the sample, with the exception of 4, had been or were being breastfed. The proportion of breastfed infants was 88% at age 3 months and 60% at age 6 months. 92% of the mothers reported that they discontinued breastfeeding at age 18 months. 

The 3rd, 5th, 10th, 15th, 25th, 50th, 75th, 85th, 90th, 95th, and 97th percentiles and the LMS values of weight and height for boys and girls are shown in Tables 1,4,respectively. 

**Figure 1:**
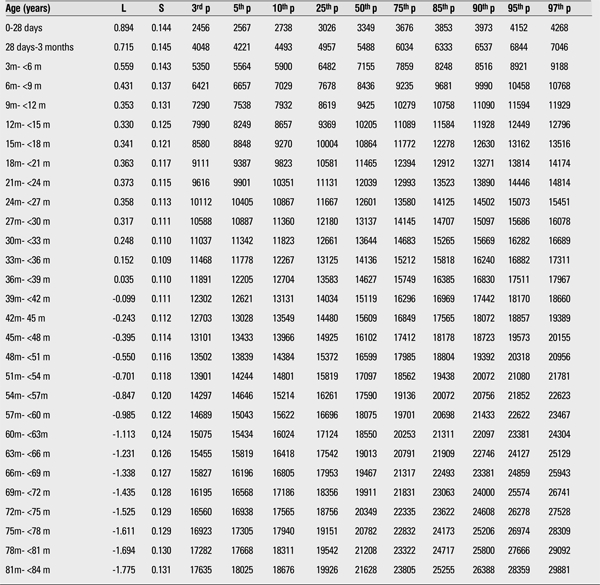
Comparison of 50th weight percentiles of Kayseri, WHO andIstanbul for boys

**Figure 2:**
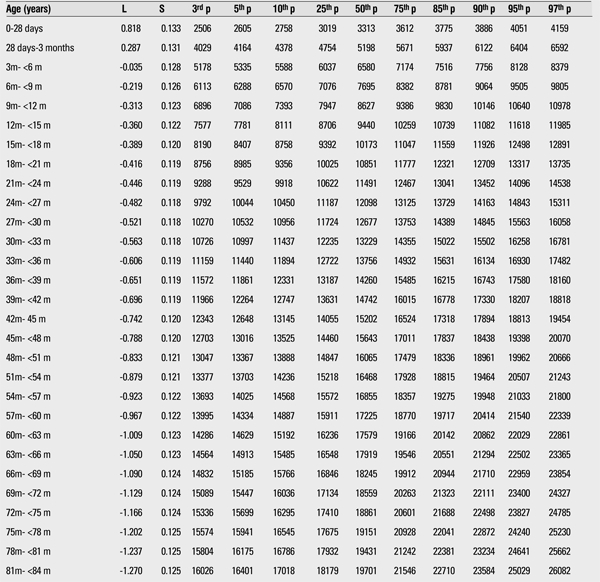
Comparison of 50th weight percentiles of Kayseri, WHO andIstanbul for girls

Figures 1 and 2 compare the 50th percentiles for weight in boys and girls of this study with WHO and Istanbul data. Weight percentiles of Kayseri children of both genders were lower in the first year when compared with the WHO standards and Istanbul references. In the 1 to 4 years age groups, weight values were slightly higher than the WHO standards and Istanbul percentiles in the girls and were higher than WHO standards, but lower than the Istanbul data in the boys (Figures 1,2). In children who were aged 4 years or older, weight percentiles of Kayseri children were higher than those of Istanbul children and WHO standards in both genders. 

**Figure 3:**
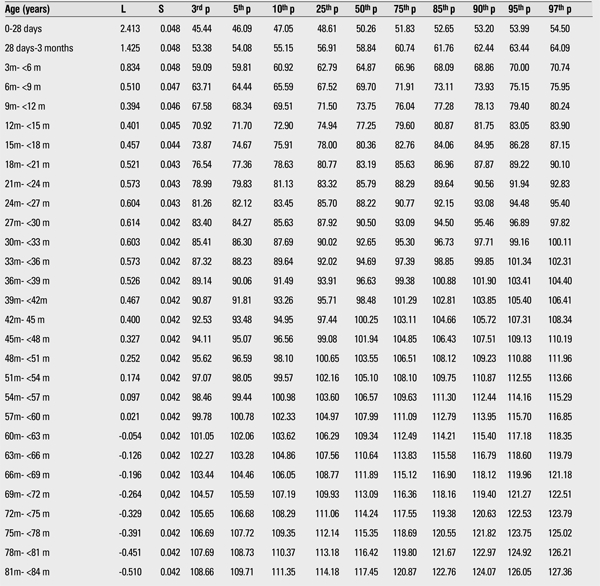
Comparison of 50th height percentiles of Kayseri, WHO andIstanbul for boys****

**Figure 4:**
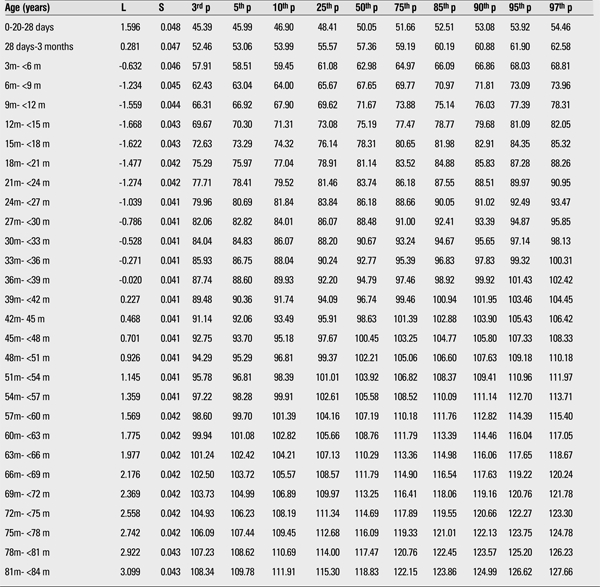
Comparison of 50th height percentiles of Kayseri, WHO andIstanbul for girls

Figures 3 and 4 compare the 50th percentiles for height in the boys and girls in this study WHO standards and Istanbul references. Height percentiles of Kayseri boys were lower in the first year and very similar in the 1 to 4 years age period when compared with the WHO standards and Istanbul references. After age 4 years, height percentiles of Kayseri boys became strikingly higher than the WHO standard and Istanbul reference values, while height percentiles of Kayseri girls were very similar to WHO standards and Istanbul references. 

We compared the height and weight of Kayseri boys and girls for 3rd, 50th, and 97th percentiles and found that boys were heavier and taller than girls. The differences at the 12-15-month age group in the 3rd, 50th, and 97th percentiles for weight were 413 g, 765 g, and 811 g, respectively (Tables 1,2). The differences in the 3rd, 50th, and 97th percentiles for height in this age group were 1.25 cm, 2.06 cm, and 1.85 cm, respectively (Tables 3,4). 

## DISCUSSION

Growth references are one of the fundamental instruments which are used in child care and deviation from growth percentiles usually reflects adverse conditions that require correction. Since the growth pattern of a population may show changes with time, these references should be updated regularly. There may also be differences in growth pattern between countries and regions (5,6,12,13,14). A study on the growth of Turkish children aged between 0 and 5 years was published in 2008. This study was longitudinal in design and the study sample consisted of infants and young children attending the Well Child Clinic of a University Hospital in Istanbul between 1992 and 2006 (15). This study provides longitudinal data from which growth rates can also be calculated. On the other hand, the present study, being cross-sectional in design, provides data on the actual status of growth in children of different ages and may be used as a basis to make future evaluations about changes in the growth status of this population (secular changes).We found that 50th percentile values for both height and weight in Kayseri children were lower than both WHO standards and Istanbul references in the first year of life (Figures 1,4). One of the explanations for this can be the difference in methodology - our study being cross-sectional in design. Various factors which may interfere with growth may also have been responsible for the difference. These can be grouped as environmental, genetic, socioeconomic, nutritional and demographic factors (16). Altitude- and microclimate-related differences have also been reported to influence growth (14,17). However, nutrition and socioeconomic status are probably the dominant factors influencing growth in the first year of life (16).We found that height percentiles of boys were similar in toddlers (1-4 years) in the Kayseri, WHO and Istanbul groups, while in girls, the height percentiles of the WHO sample were slightly higher than the Istanbul and Kayseri toddlers. The 50th percentiles for weight in Kayseri toddlers were higher than WHO standards and Istanbul references in girls, while the Kayseri boys in this age group were heavier than the boys in the WHO sample, but lower in weight as compared to the Istanbul toddlers. After infancy, nutritional influences become less important and the effect of growth hormone (GH) increasingly dominates. However, the effect of environmental, genetic and socioeconomic factors continues to be important also in this period (16).In the Kayseri sample, both boys and girls of ages 4 years or older appeared to be strikingly heavier as compared to national references and international standards. The boys were also taller, but the girls appeared to have height values comparable to those of the Istanbul children and WHO standards. Although we compared our data graphically with Istanbul references and WHO standards, we were not able make statistical comparisons since we did not have access to the original data of these studies. Our data appear to indicate that Kayseri children may be more prone to obesity than the Istanbul children, a finding which may be related to dietary habits and/or opportunities for physical exercise. However, we do not have enough data on the dietary regimens/feeding habits or lifestyle of the subjects in our sample and thus are unable to comment on the effect of these parameters on the anthropometric results. This may be a limitation of our study. In this study, we also found that boys were heavier and taller than girls. Similar gender differences in favor of boys were also observed in the national reference and international standard (5,15). In conclusion, this study shows that there are differences between our findings and national and international growth data which may be due to the effect of various factors, including dietary habits. While longitudinal growth studies provide data for the monitoring of growth, national and even local cross-sectional data will provide valuable information to evaluate the actual status of the pediatric population and serve as a basis for monitoring secular changes. 

## Figures and Tables

**Table 1:**
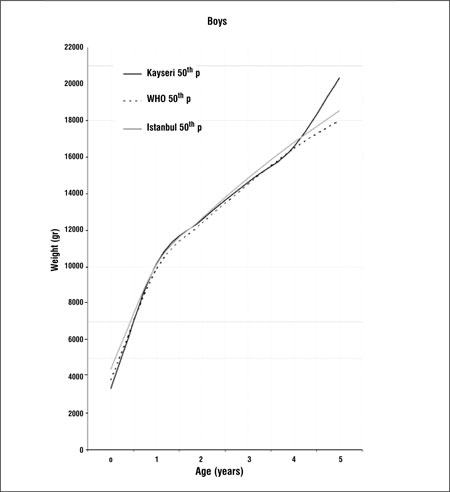
The L, S and 3rd, 5th 10th, 25th, 50th, 75th, 85th, 90th, 95th, and 97th percentiles for weight in Turkish boys

**Table 2:**
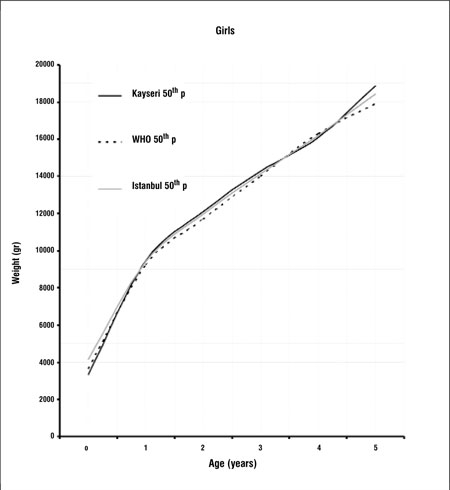
The L, S and 3rd, 5th, 10th, 25th, 50th, 75th, 85th, 90th, 95th, and 97th percentiles for weight in Turkish girls

**Table 3:**
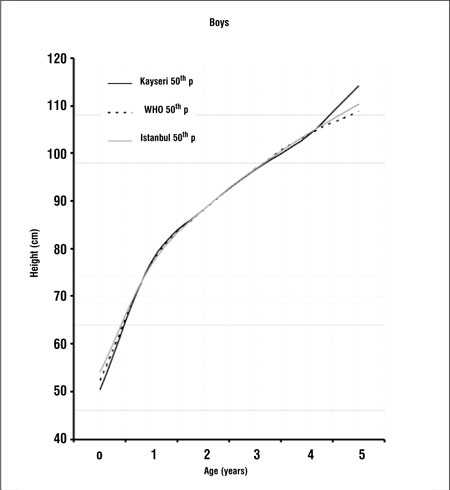
The L, S and 3rd, 5th, 10th, 25th, 50th, 75th, 85th, 90th, 95th, and 97th percentiles for height in Turkish boys

**Table 4:**
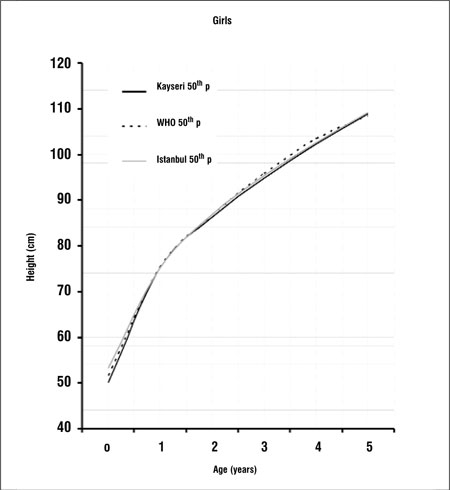
The L, S and 3rd, 5th, 10th, 25th, 50th, 75th, 85th, 90th, 95th, and 97th percentiles for height in Turkish girls
